# Predictors of drought-induced crop yield/losses in two agroecologies of southern Tigray, Northern Ethiopia

**DOI:** 10.1038/s41598-022-09862-x

**Published:** 2022-04-15

**Authors:** Emmanuel Eze, Atkilt Girma, Amanuel Zenebe, Chukwuebuka Christopher Okolo, Jean Moussa Kourouma, Emnet Negash

**Affiliations:** 1 Institute of Climate and Society, Mekelle University, Mekelle, Ethiopia; 2 Geographical and Environmental Education Unit, Department of Social Science Education, University of Nigeria, Nsukka, Nigeria; 3 Department of Land Resource Management and Environmental Protection, Mekelle University, Mekelle, Ethiopia; 4 Department of Microbiology and Ecosystem Science, Centre for Microbiology and Environmental Systems Science, University of Vienna, Althanstr. 14, 1090 Vienna, Austria; 5 Department of Plant and Environmental Science, Copperbelt University, Kitwe, Zambia; 6 Department of Geography, Ghent University, Ghent, Belgium; 7 Present Address: Institute of Geography, Faculty of Chemistry and Earth Sciences, University of Heidelberg, Heidelberg, Germany; 8 Present Address: Department of Geography - Research Group for Earth Observation (rgeo), UNESCO Chair on World Heritage & Biosphere Reserve Observation and Education, University of Education Heidelberg, Heidelberg, Germany

**Keywords:** Climate change, Natural hazards

## Abstract

The consequences of prolonged precipitation-deficient periods are primarily substantial water deficit. The spatial characteristics of drylands and various socioeconomic factors worsen droughts’ impacts and deepen poverty among agrarian communities, with attendant food security (stability dimension) implications. This study utilizes a combination of climate, remote sensing and field survey data to obtain first-hand information on the impacts of recent (2015 and 2017) droughts on crop yield in southern Tigray, northern Ethiopia. Annual and seasonal rainfall, annual and seasonal Normalized Difference Vegetation Index (NDVI) and Deviation of NDVI (Dev-NDVI), and monthly Standardized Precipitation Index (SPI) (SPI-1, SPI-3 and SPI-12) for June to October, were considered as likely factors that could relate with yield and yield loss in the area. Correlation and multiple linear stepwise regression statistical techniques were used to determine drought-yield relationships, and identify more accurate predictors of yield and yield losses in each of the drought years. The area witnessed a more widespread precipitation deficit in 2015 than in 2017, where the lowland area recorded entire crop (sorghum) losses. Also, droughts manifested spatiotemporal variations and impacts across the two different agroecologies—primarily reduction in vegetation amounts, coinciding with the planting and maturing stages of barley and sorghum. Crop failures, therefore, translated to food shortages and reduced income of smallholder farmers, which denotes food insecurity in the time of droughts. Seasonal rainfall and June Dev-NDVI predicted 66.9% of 2015 barley and sorghum yield-loss, while NDVI predicted 2017 sorghum yield by 96%. Spate irrigation should be further popularized in the low-lying areas of Raya Azebo to augment for future deficiencies in the kiremt rainfall.

## Introduction

Global climate change has resulted in the increasing occurrence of extreme weather and climatic events such as floods and droughts. According to Eze et al.^1^ and Negash et al.^2^, outputs of rain-fed agriculture are at risk of influence by drought events. Droughts occur when a shortage of precipitation is received in a given season, or when there is a departure from a known average. An abnormally prolonged dry condition is designated as drought by Bayissa et al.^3^, whereas in the view of Wilhite^4^, the timing and the effectiveness of precipitation could be linked to drought, hence making each drought event unique in impacts and characteristics. The consequences of prolonged precipitation-deficient periods are primarily substantial water deficit. Water shortages are exacerbated by the spatial characteristics of drylands, especially in arid and semi-arid environments. Additional disadvantages such as a largely rural population with very little education, totally dependent on agriculture for living and livelihood, and a limited capacity to withstand droughts could further extend impacts of droughts.

Ethiopia is a Sub-Saharan country in East Africa largely susceptible to droughts. Although some parts are more vulnerable than the other, the intensity and frequency of droughts are increasing. While Viste et al.^5^ indicate that Ethiopia is largely drought-prone, the account of Mera^6^ shows that the northern parts of Ethiopia experience droughts every 3–5 years, with a recent successive occurrence in 2015–2017. Such circumstances have been opined by Qu et al.^7^ to worsen food security issues. In describing the impacts of the most recent droughts, the Federal Democratic Republic of Ethiopia (FDRE)^8^ reports that in 2015, drought in Ethiopia was linked with the global El Niño weather phenomenon, thus resulting in food insecurity for 10.2 million people. Mera^6^ has it that the 2017 short season (March–April) rainfall was virtually a failure. Such impacts are capable of being magnified, as Gray and Mueller^9^ connect deep rural poverty with the amplification of drought effects in Sub-Saharan Africa.

Economically, droughts could deepen poverty levels especially among the largely agrarian populace of developing countries. Again, the FDRE^8^ estimates the Gross Domestic Product (GDP) losses due to climate variability to be around 1–4%. Tigray region in the northern part of Ethiopia has been identified as one of the most drought-vulnerable crop-production regions. The study of Gebrehiwot et al.^10^, classifies Tigray as one of the ‘most severely affected (regions) during well-known famines’, where a slight rainfall-deficiency can result in crop failure, with a direct impact on food security of households. Crop failure has been attributed to the paltry amount of water stored in the soil during a prolonged dry spell^11,12^. Therefore, protracted precipitation deficit to the extent of the depletion of soil moisture becomes critical for crops especially at early stages of development and maturity, with profound impacts on eventual yield in any given season^13^.

Thus, to detect and estimate droughts with their impacts, some indicators and indexes have been developed over the years. Hence, the characteristics of a drought event—onset, intensity, spatial coverage and duration, can be expressed using a drought index. In the opinion of Sivakumar^14^, parameters such as rainfall, runoff, temperature and evapotranspiration should be included in a drought index to enhance its usefulness in describing a drought event. Drought indexes have been generally grouped into meteorological, soil moisture, hydrological, remote sensing and composite/modeled indicators, to correspond with their design and scope of operations. This study is delimited to metrological and remote sensing indicators, which are described in subsequent sections. Two cereal crops—barley and sorghum, which are dominant crops and staple foods in the southern Tigray are selected for this study. These crops are hand-cultivated in rows at a small scale^15^. Animal-driven ploughs and human labour are the sources of farm power. Farmers in the chosen study area are mostly smallholders who cultivate these crops for subsistence and sell excess yield for income.

Many drought studies have focused on the assessment of both meteorological and agricultural droughts in Ethiopia. While Gebrehiwot et al.^10^ undertook a determination of spatiotemporal seasonal agricultural drought in Ethiopia during the 1998–2013 cropping seasons; Suryabhagavan^16^ characterized meteorological droughts and climate variability in Ethiopia over 3 decades (1983–2012). The study of Qu et al.^7^ on impacts of drought, includes other countries aside Ethiopia and covering the Horn of Africa, while that of Gidey et al.^17^ characterized the occurrence of drought in northern Ethiopia for 15 years (2001–2015). Only the study of Warner and Mann^18^ adopted an integrative approach of the use of satellite (GIS) data with conventional agricultural survey data to assess drought impacts, hence the essence of this study for the area.

This study rather than characterize or report drought impacts by use of indicators, or other secondary data, further utilizes the climate and remote sensing data, with field survey data from farmers to obtain first-hand information on the impacts of droughts on crop yield. We then determine climatic and vegetation drought indicators which can closely predict farmers’ yield in the recent drought events (of 2015 and 2017). As we focus on agricultural droughts in this study, we evaluate the impact of moisture deficit occurring at the critical time of the major crop growing season, which could impact crop yields adversely. Therefore, the objective and important contribution of this study to scientific knowledge of drought is to provide an insight into what drought indicators (meteorological and vegetation/remote sensing) closely relate to and predicts farmers’ crop yield, using a small geographical area, with opportunities of further discourse.

## Materials and methods

### The study area

**Figure 1:**
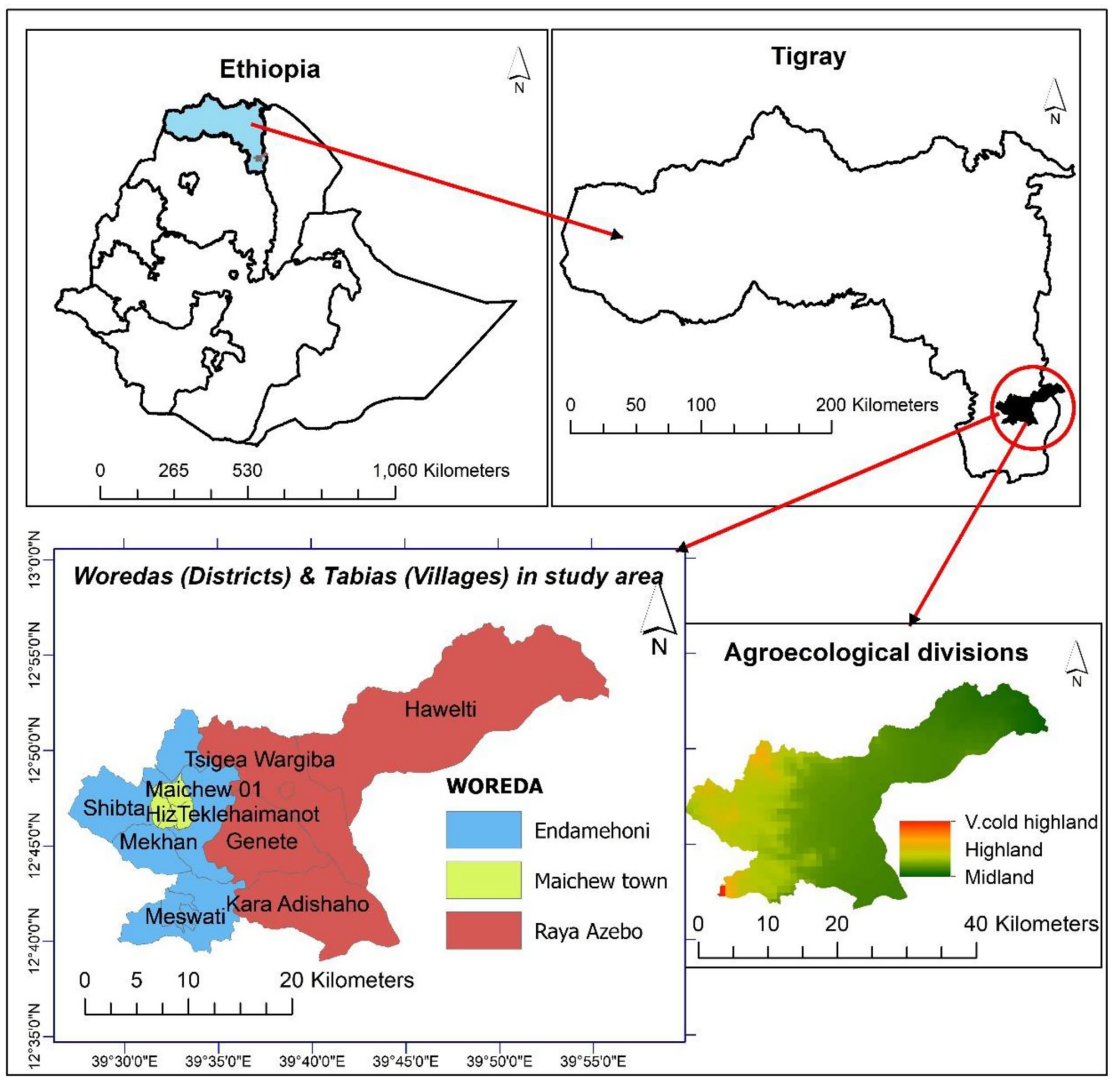
Study area map. *Source*: Authors (ArcMap 10.4).

This study was implemented in two purposively selected *woredas* (districts) in the southern Tigray region, northern Ethiopia. Tigray has been previously established as one of the most drought-vulnerable regions in Ethiopia as described in earlier sections of this paper. The study area is located between 12° 38′ 44″–12° 57′ 10″ N Latitude and 39° 27′ 18″–39° 55′ 56″ E Longitude, with an altitude ranging from 1109 to 3760 m above sea level (m.a.s.l.). The two purposively-selected woredas (districts) were to represent the highland and lowland agroecology for this study. The study area thus includes one district of mid and highland agroecology (Endamehoni) and another *woreda* (Raya Azebo) of lowland agroecology. Nine (9) *tabias/kabeles* (villages) are included in the portion selected for this study (Fig. 1).

### Data sources and analyses methods

#### Meteorological data and meteorological drought indicator

The Climate Hazards group InfraRed Precipitation with Stations (CHIRPS) grid precipitation dataset from 2015 to 2018, obtained from (ftp://ftp.chg.ucsb.edu/pub/org/chg/products/CHIRPS-2.0/) was used for this study. The CHIRPS is the combination of a “high-resolution climatology, time-varying cold cloud duration precipitation estimates, and in situ precipitation estimates”, with high research quality and very low errors^19,20^. The high reliability, spatial spread and accuracy of the CHIRPS over the point-based data makes it preferred for this study. Also, The CHIRPS has been described as dependable and accurate for use in Ethiopia, especially for obtaining data for areas without meteorological stations^21,22^. Furthermore, despite the resolution of the CHIRPS dataset being at 5 km spatial resolution, Dinku et al.^23^ conducted a validation of CHIRPS across eastern Africa and reports that the dataset offers a higher skill than other alternative rainfall products with low or no bias for Ethiopia. Moreover, Peng et al.^24^ indicate CHIRPS data as desirable for local or sub-basin scale drought monitoring studies, hence a justification for use in this study. The rainfall variables used in this study include the annual and main *(kiremt)* season (JJAS) rainfall. This data is used to assess meteorological drought using the Standardized Precipitation Index (SPI).

The commonly used meteorological drought indicators include the Standardized Precipitation Index (SPI) and the Standardized Precipitation Evapotranspiration Index (SPEI). The SPI is recommended by the World Meteorological Organization^25^ to be used by all National Meteorological and Hydrological Services around the world to characterize meteorological droughts. Designed by McKee et al.^26^ to quantify the magnitude of dry and wet conditions in the desired location, the SPI is effective in drought detection in multiple time-scales, generally between 1 and 48 months. The WMO and GWP^27^ posit that SPI values for 3 months (SPI-3) or less, might be useful for basic drought monitoring; while values for 6 months (SPI-6) or less, are fit for monitoring agricultural impacts; and values for 12 months (SPI-12) or longer, are ideal for hydrological impacts. Besides the above-mentioned points, Yildirak and Selcuk-Kestel^28^ regard SPI as easy to implement and ideal for the determination of drought risks due to its ‘intrinsic probabilistic nature’ and favourability in making spatiotemporal comparisons among diverse climatic condition. These inform the preference for SPI in this study for meteorological drought assessment. We however reiterate the limitation that since cumulative precipitation is the sole input, SPI is deficient when accounting for the temperature component of droughts, as water balance/use are not accounted for.

#### Satellite images data and agricultural drought indicators

The Moderate Resolution Imaging Spectroradiometer (MODIS) MOD13Q1 composite images with 250-m resolution data obtained from the NASA Land Processes Distributed Active Archive Center (LP DAAC), USGS/Earth Resources Observation and Science (EROS) Center (https://lpdaac.usgs.gov/data_access/data_pool), was used for this study. In the opinion of^29^, MODIS data products are accurate and appropriate for drought monitoring. Following download, the images were re-projected and resampled from a sinusoidal to geographic projection (WGS84); and then NDVI values were extracted from the re-projected (*.tif) images using R scripts. To obtain the conventional NDVI values ranging from − 1 to + 1, the extracted values were rescaled with the factor 0.0001. The NDVI variables used in this study include the Annual NDVI, the Dev-NDVI of the main cropping *(kiremt)* season, and the monthly Dev-NDVI of June to October.

A lot of satellite-based indicators have been developed, with the ability to accurately detect droughts over large spatiotemporal resolutions. The Normalized Difference Vegetation Index (NDVI) is a popular drought-monitoring index developed by Kogan^30^, for determination of droughts by the measurement of vegetation vigor and cover in an index ranging from − 1 to + 1. However, the NDVI itself needs to be computed vis-à-vis anomalies to indicate the presence of drought and its severity. The Dev-NDVI, which is the deviation of NDVI from its long-term mean will easily indicate the magnitude of wetness or dryness of an area. Hence, a negative Dev-NDVI is an indicator of below-normal vegetation condition/health, thus suggesting a drought situation. The magnitude of a drought spell is defined by the degree of negative departure from the long-term mean NDVI^31,32^. Kourouma et al.^33^ assert that ‘vegetation in general, and crops productivity, in particular, are responsive to change in NDVI values’. The NDVI and Dev-NDVI are therefore used in this study to obtain a clear perception of agricultural droughts.

#### Farmers’ yield (survey) data

Self-reported farmers’ production data were obtained from a survey of 34 smallholders for this study. A minimum N ≥ 25 is required for more variance and is recommended for regressions or meta-regressions^34^. Hence, a sample size of 34 is considered optimal for our study. A one-time direct interview of 34 farmers was conducted to elicit information on the dry grain weight of crop yield measured in quintals per hectare. After maturity, farmers harvest their grains and allow them dry in a part of the farmland. The grains are beaten for separation from their husks and then bagged. Farmers are able to weigh their grains before storage and sales.

Farm owners used in this study (sorghum farmers = 16; barley farmers = 18) were randomly selected, and are those with high yield recall from memory. Ethical procedures such as obtaining their consent to participate in the study and assurance of anonymity were strictly followed for all participants in the study. While more farmers were interviewed as they are always found in groups, only those who met our criteria of crop type and recent crop yield memory were included in the study. Their farmlands were measured with the use of HP-IPAQ (a pocket PC enabled with GIS features for plotting). The device was useful for appropriate relation of the yield to specific farmlands. Two cereal crops—barley and sorghum were selected in the highland and lowland respectively, due to their widespread importance as dominant crops and staple foods in the area, and also to improve the accuracy of yield recall from farmers’ memory.

The village *(Tabia)* or plot-level data are not available at either the Agricultural Bureau or the Central Statistics Agency. The use of district-level data available in these offices would not be representative of the local situations. This explains the choice of plot-level data in the study area, which is considered of optimum quality. Eze et al.^1^ adopted the same data to successfully develop proportionally-derived area-yield insurance index with high success in estimating payouts even for those who reported 100% losses. Also, Eze et al.^35^ attempted to develop remote crop yield estimation using crop water requirements using this data. From the evaluation of the models, there is a good fit with the survey data with a coefficient of determination (R^2^) of up to 88%. This data is thus applied in this study with the confidence of optimal performance. All methods in this study were carried out in accordance with relevant guidelines and regulations for conducting surveys. In particular, informed consent was obtained from all human subjects, and anonymity has been maintained in presenting the results obtained from analyses of collected data. We confirm that the academic board of the Institute of Climate and Society, Mekelle University, Ethiopia orally approved the conduct of this study during the presentation of the proposed research, survey instrument and method for data collection. Neither plants nor animals were used in this study, which ruled out the need for written ethical clearance or approval.

### Data analyses

#### Data pre-processing procedures

The analysis of historical meteorological droughts was done using the Standardized Precipitation Index (SPI) in R software version 3.4 with the aid of suitable scripts and packages. The SPI was calculated on three timescales: 1-, 3- and 12-month (SPI-1, SPI-3, and SPI-12). To obtain agricultural droughts Dev-NDVI was calculated using the Eq. (1).

1 $$DevNDVI = NDVI_{i} - NDVI_{mean - m}$$

where the *NDVI*_*i*_ is the NDVI value for month *‘i’*; *NDVI*_*mean-m*_ is the long-term mean NDVI for the same month m over the spatiotemporal period.

Three operations were implemented to get three variables of yield data for the analyses.

1. Using the raw yield data obtained from farmers, conversion to yield per hectare was done by dividing the actual (raw) yield value by the area of land and multiplying the result by 10,000 (which is the area of a hectare). Hence the formula:
  2 $$YpHa = \frac{Actual\, Yield}{{\overline{{Area \left( {m^{2} } \right)}} }} \times 10,000$$
  where: Actual yield = raw yield values obtained from farmers; Area(m^2^) = total area of farmer’s plot in m^2^; 10,000 = total meter squares (m^2^) in a hectare
2. The yield per hectare (YpHa) data was used to compute a Standardized variable of crop yield (SCY) for each of the sampled Tabias (villages) within the study area, using Eq. (3).
3. The SCY was then be used to obtain the crop yield loss ratio (YLR) of the area, using Eq. (4). The formulae applied to obtain the SCY and the YLR has been used in a recent study of Elhag and Zhang^36^ to monitor the impact of drought on crop yield elsewhere.
  3 $$SCY = \frac{{Yj - \overline{Y}}}{\sigma }$$
  4 $$YLR = \frac{{\overline{Y} - Yj}}{{\overline{Y}}} \times 100\%$$

where *Yj* is the crop yield in *j* Year of a *Tabia* (village); *Ȳ* is the average, and σ is the standard deviation of crop yield during the period under review.

#### Correlating yield, climatic and drought-indicator variables

To obtain the relationship between drought and crop yield in the study area, YpHa, SCY and YLR for 2015 and 2017 were correlated with annual rainfall; kiremt rainfall; annual NDVI; annual Dev-NDVI; kiremt Dev-NDVI; monthly Dev-NDVI (for June to October); and monthly SPI-1, SPI-3 and SPI-12 (for June to October). The use of all these variables in measuring the relationship is to determine which factor is most related to the declared farmers’ yield and losses for the years under review.

The analysis of the relationship between variables listed above with YpHa, SCY and YLR was done in three batches. Firstly, the relationships across the entire study area were assessed. Next, a split Woreda-based analysis was conducted to ascertain the relationships based on the two different woredas (Endamehoni and Raya Azebo) selected for the study. The purpose of the split-site analysis is to account for inherent variations expected in rainfall patterns, crop yield and crop loss in the two agroecologically dissimilar districts (*woredas*).

The non-parametric (Spearman’s) correlation analysis was implemented, as this would not demand bivariate normality of the dataset. Some previous studies^37–40^ all adopted the Spearman’s correlation for their analyses. Interpretation of the strength of association among variables as reflected by the resultant correlation coefficients adopted in this study was obtained from the groupings of Cohen et al.^41^ as shown below (Table 1).

**Table 1:** Correlation coefficients and their interpretation. *Source*: Adapted from Cohen et al.^41^.

Positive relationship		Negative relationship	
Coefficients	Strength	Coefficients	Strength
0.10–0.29	Weak/small	− 0.10 to − 0.29	Weak/small
0.30–0.49	Moderate/medium	− 0.30 to − 0.49	Moderate/medium
0.50–1.00	Strong/large	− 0.50 to − 1.00	Strong/large

#### Regressing yield, climatic and drought-indicator variables

Multiple linear stepwise regression was implemented in SPSS to obtain the most significant and relevant predictors for both crop yield and yield loss in the drought years of 2015 and 2017. The stepwise regression is an approach to selecting a subset of effects for a regression model. This approach has been widely used in literature, with several authors preferring its application where little theory to guide the selection of terms for a model exists; or where there is the focus on predictors which interactively seem to provide a good fit; and where the performance of a model is to be improved by reducing the variance from unnecessary terms^42–46^. The stepwise regression also exempts variables of high multicollinearity (variance inflation factor—VIF) in the selection of the most relevant predictors, hence its adoption in this study. The step-wise multiple regression was therefore conducted using the threshold of probability of the predictor to enter as ≤ 0.050 and the probability of the predictor to be removed as ≥ 0.100 (Table 2).

**Table 2:** Spearman’s (*r*_*s*_) correlation results of yield, drought and several climatic/vegetative indicators for the year 2015 in the study area.

Yield variables	Factors														
	ANRFE	KRFE	AND	ANDND	KRDND	Monthly DND									
						Jun	Jul	Aug	Sept	Oct					
YpHa (*r*_*s*_)	0.74**	0.75**	0.54**	0.44**	0.10	− 0.17	− 0.25	− 0.08	0.13	0.08					
SCY (*r*_*s*_)	0.71**	0.73**	0.52**	0.40*	0.08	− 0.15	− 0.32	− 0.17	0.09	0.08					
YLR (*r*_*s*_)	− 0.73**	− 0.71**	− 0.43*	− 0.31	− 0.08	0.24	0.33	0.27	− 0.10	− 0.10					

Yield variables	Factors														
	Monthly SPI-1					Monthly SPI-3					Monthly SPI-12				
	Jun	Jul	Aug	Sep	Oct	Jun	Jul	Aug	Sep	Oct	Jun	Jul	Aug	Sep	Oct
YpHa (*r*_*s*_)	− 0.55**	− 0.29	− 0.49**	0.18	− 0.59**	− 0.25	0.17	− 0.17	0.61**	− 0.29	− .52**	0.66**	0.06	0.68**	0.51**
SCY (*r*_*s*_)	− 0.51**	− 0.29	− 0.47**	0.15	− 0.56**	− 0.23	0.12	− 0.17	0.58**	− 0.27	− .43*	0.64**	0.08	0.66**	0.48**
YLR (*r*_*s*_)	0.59**	0.34*	0.51**	− 0.12	0.62**	0.37	− 0.09	0.21	− 0.51**	0.35*	0.49**	− 0.61**	0.00	− 0.59**	− 0.41*

*ANRFE* annual rainfall, *KRFE* Kiremt season rainfall, *AND* annual NDVI, *ANDND* annual dev-NDVI, *KRDND* Kiremt Dev-NDVI, *YpHa* yield (Qt) per Hectare, *SCY* standardized crop yield, *YLR* yield loss ratio, *DND* dev-NDVI.

## Results

### 2015 drought and crop yield: relationship and climatic/vegetative predictors

**Figure 2:**
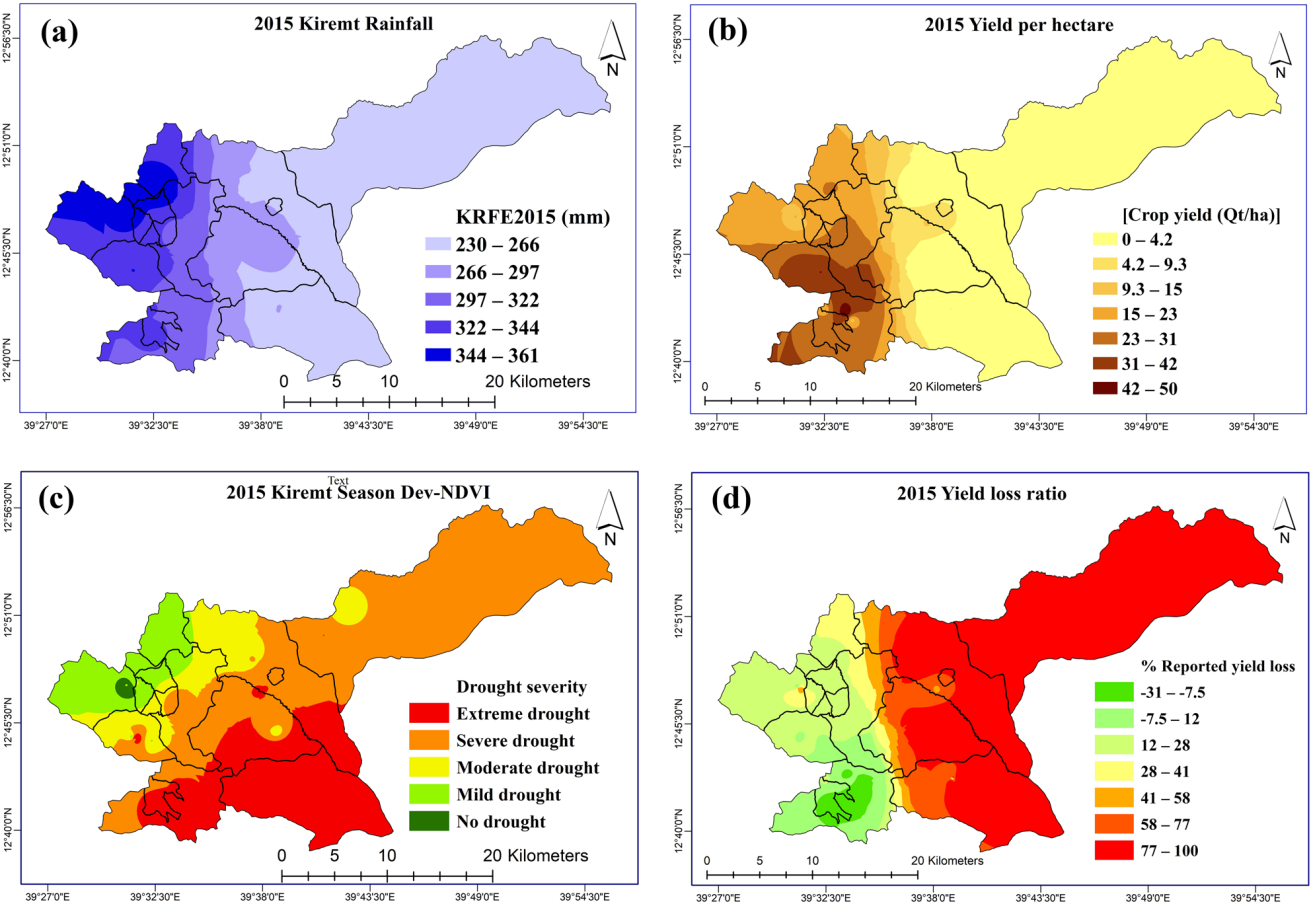
(**a**) Kiremt season rainfall (JJAS) (**b**) Farmers’ reported yield (**c**) Kiremt season Dev-NDVI, and (**d**) Farmers’ yield loss ratio for the 2015 farming season. *Source*: Authors (ArcMap 10.4).

The results are presented in three subsections to represent the lowland case study (Raya Azebo), the highland case study (Endamehoni), and the entire study area. These sections correspond to the three-batch analyses implemented to unravel the relationship of drought with crop yield and arriving at key drought indicators that more accurately predicted the crop yield in the event of drought. Figure 2 contains the key spatial variables used to obtain the results.

#### Lowland (Raya Azebo)

For the sampled area in Raya Azebo, only annual rainfall values show a strong positive significant correlation with SCY and YpHa, while June and October SPI-1 indicate a strong negative association with SCY and YpHa (Table 3). This implies that higher annual rainfall translates to higher crop yield while higher meteorological drought in June and September led to lower crop yield. *Kiremt* season rainfall also had a strong positive significant relationship with YpHa. Other notable variables indicating a moderate positive relationship with YpHa and SCY are annual Dev-NDVI, *kiremt* Dev-NDVI; while a moderate negative relationship exists between July and August SPI-1, June and July SPI-1; June and October SPI-3 (Table 3). Annual rainfall and *kiremt* season rainfall had a strong negative relationship with YLR. Hence, the lower the (annual and *kiremt*) rainfall, the higher the yield loss. On the other hand, annual Dev-NDVI, June and October SPI-1 are strongly positively and significantly associated with YLR. Hence, a higher deviation from NDVI and higher meteorological drought reflected in higher crop losses. *Kiremt* Dev-NDVI, June Dev-NDVI, July SPI-1, June and October SPI-3, and June SPI-12 were moderately positively correlated with YLR.

**Table 3:** Spearman’s (*r*_*s*_) correlation results of yield, drought and several climatic/vegetative indicators for the year 2015 in Raya-Azebo.

Yield variables	Factors														
	ANRFE	KRFE	AND	ANDND	KRDND	Monthly DND									
						Jun	Jul	Aug	Sep	Oct					
YpHa (*r*_*s*_)	0.52*	0.59*	− 0.01	− 0.42	− 0.39	− 0.22	− 0.15	− 0.00	0.06	0.09					
SCY (*r*_*s*_)	0.52*	0.45	− 0.18	− 0.36	− 0.34	− 0.19	− 0.23	− 0.15	0.07	0.06					
YLR (*r*_*s*_)	− 0.52*	− 0.57*	0.18	0.51*	0.47	0.34	0.01	0.12	0.00	0.09					

Yield variables	Factors														
	Monthly SPI-1					Monthly SPI-3					Monthly SPI-12				
	Jun	Jul	Aug	Sep	Oct	Jun	Jul	Aug	Sep	Oct	Jun	Jul	Aug	Sep	Oct
YpHa (*r*_*s*_)	− 0.52*	− 0.35	− 0.39	− 0.13	− .52*	− 0.43	− 0.13	− 0.13	0.05	− 0.43	− 0.37	0.41	− 0.08	0.33	− 0.02
SCY (*r*_*s*_)	− 0.51*	− 0.39	− 0.47	− 0.29	− .51*	− 0.43	− 0.29	− 0.36	− 0.06	− 0.43	0.00	0.24	− 0.15	0.21	− 0.20
YLR (*r*_*s*_)	0.52*	0.43	0.27	0.219	0.52*	0.48	0.22	0.03	0.08	0.48	0.36	− 0.37	0.08	− 0.21	0.14

Annual rainfall and July SPI-3 are the most significant predictors of SCY in Raya Azebo. The two-step regression (Supplementary Table S.01) results show that annual rainfall alone explains 38% of the variation in the standardized crop yield while and the combination of both predictors—annual rainfall and July SPI-3 for the year explain 56.3% of the variation in SCY for Raya Azebo (Table 5). Similar to the SCY, the YpHa is significantly predicted by annual rainfall and July SPI-3 with the coefficient of determination (*r*^*2*^) indicating that 35.7% of the variations in the yield per hectare obtained in the study area is attributed to annual rainfall alone, while the combination of annual rainfall and July SPI-3 explains 53.3% of the variations (Table 5). For the 2015 season, about 75% of farmers sampled in Raya Azebo had zero yields and 100% of crop losses. The stepwise regression for YLR was unsuccessful due to the multiple zero figures.

#### Highland (Endamehoni)

There are slightly different results obtained in the analyses of data in this area. There is a strong negative significant relationship between *kiremt* Dev-NDVI, June Dev-NDVI, July and September SPI-1 and SPI-3; and July September and October SPI-12, all with YpHa (Table 4). The SCY had a strong negative significant relationship with kiremt Dev-NDVI, September SPI-1; July and September SPI-3; and July, September and October SPI-12. Additionally, it is worthy of mention that the correlation between *kiremt* season rainfall, August SPI-1, June, August and October SPI-3, and August SPI-12 with YpHa and SCY was moderately negative, though not significant. Therefore, higher wetness led to lower yield in this *woreda*. The crop yield loss was strongly and positively significantly related with Annual Dev-NDVI, June Dev-NDVI, August and September SPI-1; June to October SPI-3, and August to October SPI-12. Furthermore, *kiremt* season rainfall, July SPI-1 and July SPI-12 showed a moderate positive relationship with the YLR but not significant. Higher rainfall reflected in higher yield loss in this area and higher Dev-NDVI did not imply higher yield as crop losses were recorded due to excessive rainfall, as confirmed by data obtained during farmers’ survey (Table 4).

**Table 4:** Spearman’s (*r*_*s*_) correlation results of yield, drought and several climatic/vegetative indicators for the year 2015 in Endamehoni.

Yield variables	Factors														
	ANRFE	KRFE	AND	ANDND	KRDND	Monthly DND									
						Jun	Jul	Aug	Sep	Oct					
YpHa (*r*_*s*_)	0.056	− 0.461	− 0.333	− 0.162	− .577*	− 0.490*	0.358	0.361	0.224	0.04					
SCY (*r*_*s*_)	− 0.088	− 0.439	− 0.201	− 0.272	− .567*	− 0.385	0.16	0.163	0.067	0.035					
YLR (*r*_*s*_)	− 0.189	0.389	0.366	0.414	.483*	0.409	0.173	0.144	− 0.245	− 0.251					

Yield variables	Factors														
	Monthly SPI-1					Monthly SPI-3					Monthly SPI-12				
	Jun	Jul	Aug	Sep	Oct	Jun	Jul	Aug	Sep	Oct	Jun	Jul	Aug	Sep	Oct
YpHa (*r*_*s*_)	− 0.13	− 0.55*	− 0.44	− 0.62**	− 0.13	− 0.44	− .58*	− 0.44	− .52*	− 0.44	− 0.27	− 0.51*	− 0.44	− 0.52*	− 0.59*
SCY (*r*_*s*_)	0.02	− 0.43	− 0.30	− 0.52*	0.02	− 0.30	− .51*	− 0.30	− .53*	− 0.30	− 0.12	− 0.49*	− 0.30	− 0.53*	− 0.52*
YLR (*r*_*s*_)	0.17	0.47	.49*	0.50*	0.17	0.49*	0.57*	0.49*	0.55*	0.49*	0.10	0.45	.049*	0.551	0.61**

*ANRFE* annual rainfall, *KRFE* Kiremt season rainfall, *AND* annual NDVI, *ANDND* annual dev-NDVI, *KRDND* Kiremt Dev-NDVI, *YpHa* yield (Qt) per Hectare, *SCY* standardized crop yield, *YLR* yield loss ratio, *DND* dev-NDVI.

October SPI-12 is the most significant predictor of SCY in Endamehoni for 2015. The coefficient of determination (*r*^*2*^) shows that October SPI-12 explain 43.7% of the variation in standardized crop yield in the area for the year under review (Table 5). For the YpHa, October SPI-12 and August Dev-NDVI are the key predictors, with 42.2% of the variations in the yield per hectare obtained in the study area attributed to October SPI-12 alone, while the combination of August Dev-NDVI with October SPI-12 explains 56.9% of the variations in yield per hectare. A two-step regression (Supplementary Table S.01) indicates September SPI-12 and October Dev-NDVI as the key predictors of yield loss. The coefficient of determination (*r*^*2*^) show that September SPI-12 alone explains 38.4% of the variation in yield loss ratio; while the combination of September SPI-12 and October Dev-NDVI explain 62.4% (Table 5).

**Table 5:** Predictors of YpHa, SCY and YLR in the 2015 drought year in the study area.

Location	Variable	Highest regression step	Predictors	VIF	R	R^2^	Sig
Raya Azebo	YpHa	2	Annual_RFE, July15_SPI3	2.953 2.953	.730	.533	.003** .045*
	SCY	2	Annual_RFE, July15_SPI3	2.953 2.953	.750	.563	.002** .037*
	YLR	Nil					
Endamehoni	YpHa	2	oct15_SPI12, Aug15_DNDVI	1.014 1.014	.754	.569	.001*** .039*
	SCY	1	oct15_SPI12	1.000	.661	.437	.003*
	YLR	2	sep15_SPI12, oct15_DNDVI	1.014 1.014	.790	.624	.001*** .007**
Entire area	YpHa	1	Annual_RFE	1.000	.635	.403	.000***
	SCY	3	KRFE2015, jun15_DNDVI, ND15	1.731 1.217 1.997	.758	.575	.004** .002** .003**
	YLR	2	KRFE2015, jun15_DNDVI	1.049 1.049	.818	.669	.000*** .001***

***Regression predictor is significant at the above 0.001 level.

**Regression predictor is significant at the 0.01 level.

*Regression predictor is significant at the 0.05 level.

#### Entire study area

From the results obtained from the entire area analysis, there is a strong positive significant relationship between Annual Rainfall and Kiremt Rainfall with farmers’ YpHa and SCY (Table 2). This implies that higher rainfall led to higher crop yield. Conversely, there is a strong negative significant relationship between annual rainfall, kiremt rainfall and farmers’ YLR. Besides, the annual NDVI shows a strong positive significant relationship between YpHa and SCY while annual Dev-NDVI shows a moderate/medium positive significant relationship between the same variables. This implies that the deviation of vegetation index from the long-term average does not always translate to yield reduction. Farmers’ YLR shows a moderate negative significant relationship with Annual NDVI and a weak negative relationship with Annual Dev-NDVI (Table 2).

Furthermore, the monthly Dev-NDVI indicate various relationships with farmers’ YpHa, SCY and YLR. The SPI at the 1, 3, and 12-month scales show different levels of relationship for different months with the YpHa, SCY and YLR. At the 1-month scale of SPI, there is a strong negative significant relationship between YpHa, SCY and SPI-1 values for June and October, with August showing a nearly strong negative significant association (Table 2). This implies that higher SPI-1 drought during the planting and maturing stages impacted crops’ performance and resulted in yield reduction. A strong positive significant relationship exists between YLR and SPI-1 for June, August and October meaning that higher droughts in these months, which are crucial growing and maturing phases of the crops studied, translates to higher yield losses. The 3-month scale of SPI shows a strong positive significant relationship with YpHa and SCY in September and a strong negative significant relationship with YLR. For the 12-month scale of SPI, a strong positive significant relationship exists between YpHa and SCY in July, September and October (Table 2). While the YLR has a strong negative significant relationship with July and September SPI-12, it has a medium negative significant relationship with October SPI-12 values (Table 2). Hence, higher SPI-12 drought in July and September led to higher crop losses, whereas October drought had no major impact on crop losses.

*Kiremt* season rainfall, June Dev-NDVI and annual NDVI are the key predictors for SCY in the entire study area. From the three-step regression, *kiremt* rainfall alone explains 39.8% of the variation in the standardized crop yield; *Kiremt* rainfall and June Dev-NDVI explain 50.4%; and the combination of all three predictors—*kiremt* rainfall, June Dev-NDVI and the annual NDVI for the year explain 57.5% of the variation in crop yield in the study area. The key predictors for YLR are *kiremt* rainfall and June Dev-NDVI. The two-step regression shows that *kiremt* rainfall alone explains 51.3% of the variation in yield loss ratio; while the combination of *kiremt* rainfall and June Dev-NDVI explain 66.9% (Table 5). Some additional variables that are excluded from the step-wise threshold, though with significant predictive possibilities but high multicollinearity, include the September SPI-3 (*p* = 0.047) and October SPI-12 (*p* = 0.046). From a one-step regression analysis (Supplementary Table S.01), annual rainfall is the most significant predictor of YpHa. The coefficient of determination (*r*^*2*^) obtained shows that 40.3% of the variations in the yield per hectare obtained in the entire study area is attributed to annual Rainfall.

### 2017 drought and crop yield: relationship and climatic/vegetative predictors

**Figure 3:**
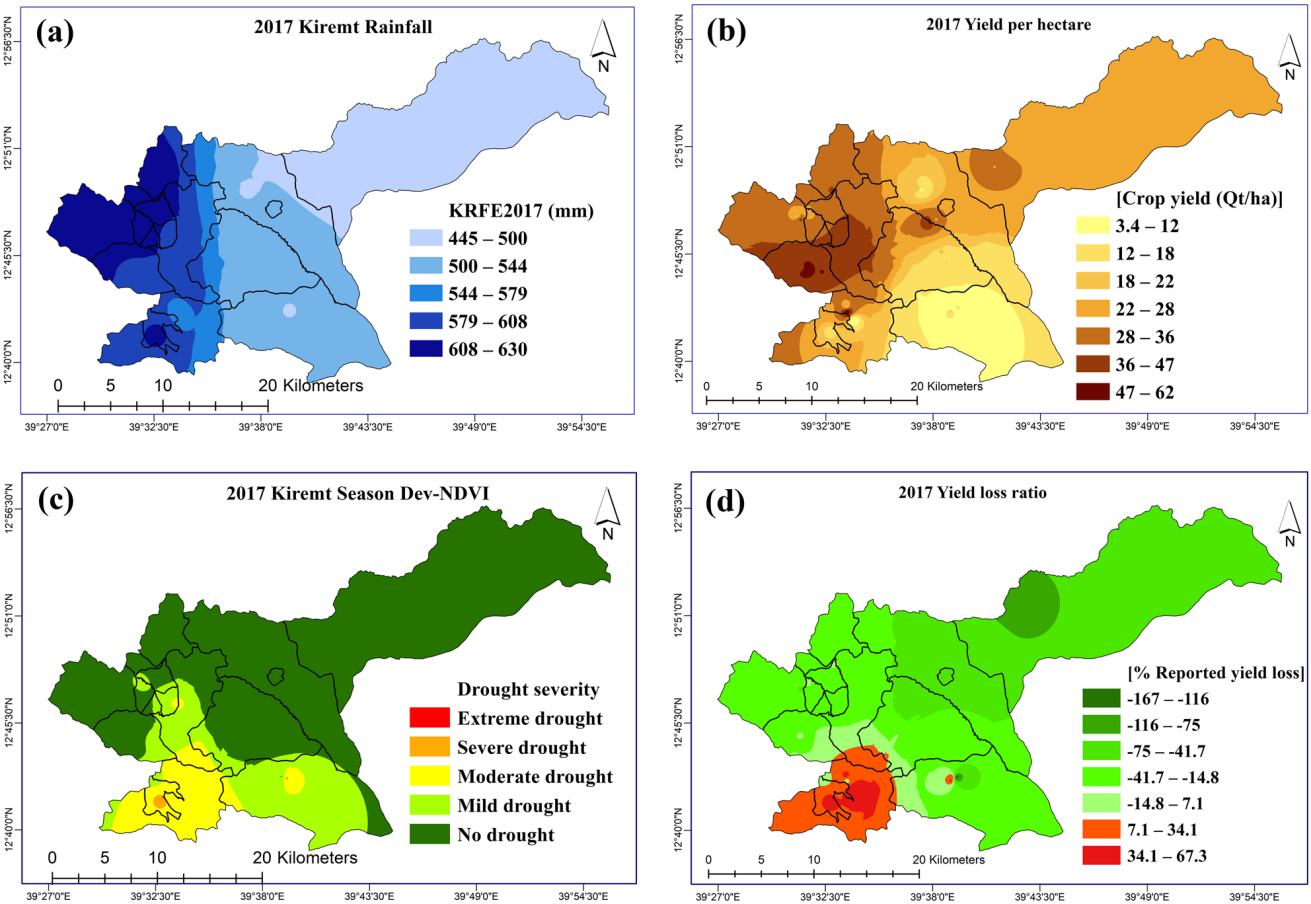
(**a**) Kiremt season rainfall (JJAS) (**b**) Farmers’ reported yield (c) Kiremt season Dev-NDVI, and (**d**) Farmers’ yield loss ratio for the 2017 farming season. Source: Authors (ArcMap 10.4).

The pattern observable (Fig. 3) is inconsistent with the 2015 drought year (Fig. 2) as no location recorded 100% crop loss. Also, areas that had higher crop losses in the previous drought of 2015 had better and improved yield in this season. As earlier indicated above, the results are presented in three subsections to represent the lowland case study (Raya Azebo), the highland case study (Endamehoni), and the entire study area. These sections also correspond to the three-batch analyses implemented to unravel the relationship of drought with crop yield and arriving at key drought indicators that more accurately predicted the crop yield in the drought events (Table 6).

**Table 6:** Spearman’s (*r*_*s*_) correlation results of yield, drought and several climatic/vegetative indicators for the year 2017 in the study area.

Yield variables	Factors														
	ANRFE	KRFE	AND	ANDND	KRDND	Monthly DND									
						Jun	Jul	Aug	Sep	Oct					
YpHa (*r*_*s*_)	0.37*	0.38*	0.39*	0.00	0.13	− 0.07	− 0.12	− 0.05	− 0.08	− 0.01					
SCY (*r*_*s*_)	0.26	0.29	0.45**	0.07	0.22	-0.03	− 0.15	− 0.08	− 0.10	− 0.00					
YLR (*r*_*s*_)	0.41*	0.35*	− 0.24	− 0.40*	− 0.50**	− 0.14	0.19	− 0.15	− 0.08	− 0.05					

Yield variables	Factors														
	Monthly SPI-1					Monthly SPI-3					Monthly SPI-12				
	Jun	Jul	Aug	Sep	Oct	Jun	Jul	Aug	Sep	Oct	Jun	Jul	Aug	Sep	Oct
YpHa (*r*_*s*_)	− 0.15	0.39*	− 0.11	0.44**	0.41*	− 0.10	0.04	− 0.12	0.46**	0.43*	− 0.45**	0.47**	− 0.18	0.33	0.46**
SCY (*r*_*s*_)	− 0.07	0.36*	− 0.05	0.41*	0.36*	− 0.03	0.07	− 0.07	0.37*	0.36*	− 0.37*	0.38*	− 0.11	0.28	0.37*
YLR (*r*_*s*_)	− 0.21	− 0.1	− 0.14	− 0.17	− 0.00	− 0.38*	− 0.24	− 0.07	0.25	0.25	− 0.11	0.31	0.05	0.13	0.30

#### Raya Azebo (lowland)

There is a strong positive significant relationship between annual NDVI with YpHa and SCY. A positive moderate not-significant relationship also exists between *kiremt* Dev-NDVI, YpHa and SCY (Table 7). June to September Dev-NDVI values show a moderate negative not-significant relationship with YpHa and SCY. Crop loss relates moderately with *kiremt* season rainfall, June and July Dev-NDVI, August SPI-1; July to September SPI-12 values. All these were not significant in their relationship. However, June SPI-3 has a strong negative significant relationship with YLR while October SPI-12 has a strong positive relationship with YLR, which is also significant (Table 7).

**Table 7:** Spearman’s (*r*_*s*_) correlation results of yield, drought and several climatic/vegetative indicators for the year 2017 in Raya Azebo.

Yield variables	Factors														
	ANRFE	KRFE	AND	ANDND	KRDND	Monthly DND									
						Jun	Jul	Aug	Sep	Oct					
YpHa (*r*_*s*_)	− 0.32	− 0.01	0.66**	0.35	0.53*	− 0.30	− 0.49	− 0.43	− 0.43	− 0.11					
SCY (*r*_*s*_)	− 0.32	− 0.01	0.67**	0.35	0.53*	− 0.30	− 0.48	− 0.42	− 0.42	− 0.13					
YLR (*r*_*s*_)	0.35	0.46	− 0.17	0.02	− 0.13	0.51*	0.61*	0.20	0.17	0.07					

Yield variables	Factors														
	Monthly SPI-1					Monthly SPI-3					Monthly SPI-12				
	Jun	Jul	Aug	Sep	Oct	Jun	Jul	Aug	Sep	Oct	Jun	Jul	Aug	Sep	Oct
YpHa (*r*_*s*_)	0.37	0.32	0.21	0.59*	0.32	.508*	0.01	0.16	0.04	0.15	− 0.2	0.02	0.07	0.00	0.09
SCY (*r*_*s*_)	0.37	0.32	0.21	0.59*	0.32	.508*	0.01	0.16	0.04	0.15	− 0.2	0.02	0.07	0.00	0.09
YLR (*r*_*s*_)	− 0.06	0.00	0.18	− 0.23	0.002	− 0.50*	− 0.32	0.228	0.327	0.203	0.18	0.461	0.327	0.38	.507*

The results of a seven-step multiple regression (Supplementary Table S.02) indicate that annual NDVI for 2017, and June to October Dev-NDVI values are key predictors of SCY. The combination of annual NDVI, June Dev-NDVI, August Dev-NDVI, July Dev-NDVI and October Dev-NDVI explains 96% of the variation of SCY in Raya Azebo for this farming season. Similarly, a seven-step regression presents the annual NDVI and Dev-NDVI, June SPI-12, June, August and September Dev-NDVI as the most significant predictors of YpHa with 96.6% explanation of the variation in yield per hectare obtained in Raya Azebo (Table 9). The key predictor of crop losses in Raya Azebo during the 2017 drought was Kiremt Rainfall (Table 9). The coefficient of determination (*r*^*2*^) obtained shows that 37.1% of the variations in the yield loss ratio obtained in the study area in 2017 is attributed to the kiremt rainfall values (Table 9).

#### Endamehoni (highland)

Key results found in this area indicate that YpHa and SCY are moderately positively correlated with Annual and June Dev-NDVI (Table 8). The SCY, however, shows a strong positive relationship with *kiremt* Dev-NDVI, August and September Dev-NDVI. The YLR had a strong negative significant relationship with *kiremt* Dev-NDVI, September SPI-3; July, September and October SPI-12. The variable also had a moderate negative correlation with annual and June Dev-NDVI; July, September and October SPI-1; October SPI-3 values.

**Table 8:** Spearman’s (*r*_*s*_) correlation results of yield, drought and several climatic/vegetative indicators for the year 2017 in Endamehoni.

Yield variables	Factors														
	ANRFE	KRFE	AND	ANDND	KRDND	Monthly DND									
						Jun	Jul	Aug	Sep	Oct					
YpHa (*r*_*s*_)	0.02	− 0.17	0.13	0.11	0.23	0.30	0.29	0.54*	0.58*	0.16					
SCY (*r*_*s*_)	− 0.05	− 0.12	0.30	0.14	0.28	0.39	0.26	0.43	0.43	0.18					
YLR (*r*_*s*_)	− 0.00	− 0.38	− 0.42	− 0.32	− 0.59*	− 0.55*	− 0.15	− 0.14	0.05	0.09					

Yield variables	Factors														
	Monthly SPI-1					Monthly SPI-3					Monthly SPI-12				
	Jun	Jul	Aug	Sep	Oct	Jun	Jul	Aug	Sep	Oct	Jun	Jul	Aug	Sep	Oct
YpHa (*r*_*s*_)	− 0.16	0.10	− 0.13	− 0.04	− 0.06	− 0.12	0.19	− 0.13	0.02	− 0.10	− 0.40	0.07	− 0.18	− 0.7	0.04
SCY (*r*_*s*_)	− 0.09	0.12	− 0.02	0.02	− 0.00	− 0.06	0.15	− 0.02	0.07	− 0.01	− 0.28	0.10	− 0.08	0.8	0.08
YLR (*r*_*s*_)	0.07	− 0.44	− 0.19	− 0.42	− 0.45	0.02	− 0.00	− 0.19	− .60**	− 0.45	0.041	− 0.57*	0.09	− 0.52*	− 0.50*

*ANRFE* annual rainfall, *KRFE* Kiremt season rainfall, *AND* annual NDVI, *ANDND* annual dev-NDVI, *KRDND* Kiremt dev-NDVI, *YpHa* yield (Qt) per hectare, *SCY* standardized crop yield, *YLR* yield loss ratio, *DND* dev-NDVI.

**Correlation is significant at the 0.01 level (2-tailed).

*Correlation is significant at the 0.05 level (2-tailed).

The most relevant predictors of SCY in Endamehoni are September Dev-NDVI and the 2017 Kiremt Dev-NDVI in a two-step computation (Supplementary Table S.02). The coefficient of determination (*r*^*2*^) shows that only the September Dev-NDVI is a predictor of SCY explain 29.5% of the variation, while the addition of the kiremt season Dev-NDVI for the year explains 46.9% variation in standardized crop yield in the area for the year under review (Table 9). Similarly, the September Dev-NDVI is the sole predictor of YpHa in the area with 45.2% of variations explained in the one-step regression implemented (Table 9). September SPI-3 is the only, most relevant and significant predictor of crop losses in this location for the year 2017. The coefficient of determination (*r*^*2*^) shows that September SPI-3 explains 45.4% of the variation in yield loss ratio in the area (Table 9). A nearly-significant predictor is the June Dev-NDVI with (*p* = 0.052).

**Table 9:** Predictors of YpHa, SCY and YLR in the 2017 drought year in the study area.

Location	Variable	Highest regression step	Predictors	VIF	R	R^2^	Sig
Raya Azebo	YpHa	7	sep17_DNDVI, jun17_DNDVI, aug17_DNDVI, june17_SPI12, DNY2017	2.622 1.191 2.308 1.382 1.755	.983	.966	.027* .000*** .004** .000*** .000***
	SCY	7	ND17, jun17_DNDVI, aug17_DNDVI, jul17_DNDVI, oct17_DNDVI	1.587 1.678 1.624 2.068 1.553	.980	.960	.000*** .000*** .000*** .009** .029*
	YLR	1	KRFE2017,	1.000	.609	.371	.012*
Endamehoni	YpHa	1	Sep17_DNDVI	1.000	.672	.452	.002**
	SCY	2	sep17_DNDVI, KSDN17	1.013 1.013	.685	.469	.007** .043*
	YLR	1	sep17_SPI3	1.000	.674	.454	.002**
Entire area	YpHa	1	Jul17_SPI12	1.000	.494	.244	.003**
	SCY	2	ND17, Jul17_SPI12	1.028 1.028	.575	.331	.015* .018*
	YLR	2	KRFE2017, sep17_SPI3	7.822 + 7.822	.575	.330	.002** .016*

***Regression predictor is significant at the above 0.001 level.

**Regression predictor is significant at the 0.01 level.

*Regression predictor is significant at the 0.05 level.

#### Entire study area

The results depict a moderate positive significant relationship between annual rainfall, kiremt season rainfall, annual NDVI and YpHa (Table 6). Similarly, a moderate positive significant relationship exists between Annual NDVI and SCY. Also, the July, September and October SPI-1; September and October SPI-3; together with June, July and October SPI-12 all show a moderate positive relationship with YpHa and SCY. In correlating crop loss with the various factors, only the annual rainfall and kiremt season rainfall showed a positive significant relationship with YLR, while June SPI-3 values show a moderate positive significant relationship.

Annual NDVI and July SPI-12 are the key predictors of SCY from the two-step multiple regression analysis implemented (Supplementary Table S.02). Annual NDVI alone explains 19.6% of the variation in the standardized crop yield while the combination of both predictors—annual NDVI and the July SPI-12 values for the year explain 33.1% of the variation in SCY (Table 9). However, for the YpHa, July SPI-12 as the only and most relevant and significant predictor with the coefficient of determination (*r*^*2*^) obtained shows that 24.4% of the variations in the yield per hectare obtained in the study area is attributed to the July SPI-12 values (Table 9).

The most significant predictors of yield loss in the study area are kiremt rainfall and September SPI-3. The coefficient of determination (*r*^*2*^) show that kiremt rainfall alone explains 19% of the variation in yield loss ratio; while the combination of kiremt rainfall and September SPI-3 explain 33% variations in yield loss within the entire study area (Table 9).

## Discussion

The year 2015 is adjudged as the most recent drought year of high magnitude and one of the worst events over the past 30 years covering a large area and exacerbating food insecurity in Ethiopia^3,47^. These literature reports are supported by evidence of low crop yield in a larger part of the study area (Fig. 2). Also, the year 2017 is recorded as the most recent drought year in some parts of Tigray/Ethiopia with Gross Domestic Product (GDP) losses due to climate variability estimated to be around 1–4% excluding human losses^8^. These events were expected to have impacted on crop yield and yield losses in the study area.

From this study, drought has again been proven to be location-specific, and not a broad spatio-temporal phenomenon at any given time. In an earlier study, Viste et al.^5^ describe most historic droughts as being more of ‘local or regional character’ with dissimilar effects at different places and seasons. Each drought year reviewed (2015 and 2017) stands out uniquely in its nature and characteristics. The only common denominator is precipitation-deficit, which in itself may be of different magnitude per location. Overall, the seasonal rainfall benchmark of 500 mm as stipulated by Haile et al.^48^ for a rough drought indicator in a season helps to present an outlook of the drought. In 2015, the entire study area fell below the 500 mm mark, while in 2017, only one *tabia/kabele* (village) did. Hence the 2015 drought was more widespread than that of 2017, with the majority of farmers in the lowland reporting entire crop losses. The predictors of yield and yield losses indicate that droughts coinciding with planting and maturing stages of crops are critical and inimical to crop production and agricultural sustainability and food security.

Specifically, the stability (time) dimension of food security is of concern in this study. According to the definition of Food and Agriculture Organization of the United Nations^49^ the stability dimension also referred to as the time dimension of food security by Peng and Berry^50^ refers the ability of a person, community or nation to withstand food system shocks arising from either natural or man-made disaster sources. The widespread crop losses witnessed in the lowland communities due to the 2015 drought indicate the susceptibility of their food systems to drought events. This condition requires the adoption of preventive adaptation measures and improved preparedness to forestall future occurrences on the impact. It is necessary to strengthen the stability dimension of food security of the vulnerable communities because of its potential effect on other levels and dimensions of food security^50^.

In both years (2015 and 2017), and for the entire study area, both annual and the kiremt seasonal rainfall were related to the crop yield reported by farmers. Crop yield is largely a function of adequate soil moisture at all stages of crop growth and maturity. Hence, the shortage of rainfall (occurrence of drought) brought about reduced crop yield in the area. These assertions are corroborated by the findings of Gidey et al.^17^ who reported that agricultural drought responds positively to seasonal rainfall. Also, part of the findings of previous studies^51–53^ indicate that rainfall significantly determines the occurrence of drought, hence manifesting high influence on the growth and development of vegetation.

Different timescales of SPI, especially the 3- and 12-month scales calculated in this study were recurring predictors of crop yield in the area. Although, the 6-month scale is excluded in this study, we can infer that it is likely to be a predictor of drought. In relating meteorological drought index for drought monitoring, several studies have found a significant relationship between SPI and crop yield^54–56^. Also, the vegetation anomaly (Dev-NDVI) can detect agricultural drought and predict crop yield in the area. These rainfall and vegetation based indices used in this study considered vis-à-vis specific crop timelines are helpful to clarify the periods drought were more critical. For example, the SPI-3 for July 2015 predicted sorghum yield for the lowland portion of the study area, because sorghum crops are at their early stages of growth at this particular time. In the mid and highland areas, the Dev-NDVI for October 2015 predicted yield and yield loss as the late maturity period for barley includes October. Hence, the satellites detected low vegetation vigor, indicating drought at the time. For the Dev-NDVI to detect agricultural drought in October, it also implies that preceding moisture deficit has occurred.

The reduced impact of drought on crop yield in 2017, especially in the lowland area is attributable to the increased rainfall received. Most of the *tabias* (villages) received rainfall higher than 500 mm, which supports healthy and sustainable agriculture. Additionally, the report of Madajewicz et al.^57^ contains Oxfam’s contribution to the minimization of drought risks by the construction of canals covering 862 hectares of land to supplement rain-fed agriculture in the occurrence of a drought event, pointing to the increased popularity of spate irrigation systems. This may likely have been utilised in these areas resulting in reversing the report of 100% crop losses witnessed in 2015, and obtaining above-normal crop yield in 2017, despite droughts. Steenbergen et al.^58^ describe the dual benefit of spate irrigation as being a means of both soil and water conservation. Hence, while water channeled from the highlands to the midlands and lowlands are stored for use as required, the alluvial deposit that accompanies the flood are rich in nutrients to improve crop yields and reduce costs of purchasing fertilizers^58^.

## Conclusion

This study critically assessed the recent drought years (2015 and 2017) in northern Ethiopia, with a focus on a small area containing high, mid and lowland agroecology. Results obtained show droughts as having spatiotemporal variations and impacts, with its primary and common reflection being reduced vegetation amount. This translated to crop failures, the reduced income of smallholders, food shortages and food insecurity. SPI, NDVI and Dev-NDVI were found quite useful as indicators of drought and considered able to be applied in the preparation of early warning for droughts when they fall below a certain established threshold for the area in each cropping season, especially at critical crop growth and maturity stages. Findings of this study can provide information for further discussions in improving drought monitoring at lower locational scales. The stability (time) dimension of food security was affected by the 2015 drought in the lowland areas as most farmers report entire sorghum losses. Irrigation channels to flow from the highland areas to the lowland areas (spate irrigation) could be popularized as an adaptation measure to reduce vulnerability to droughts. This will improve food security/stability in the face of climatic events such as droughts by providing supplementary water supply to meet the crops’ water requirements especially during the growing and maturity periods. With the provision of supplementary water source(s) during rainfall shortages in cropping seasons, the impacts of future droughts on food security are reduced. There could be more factors responsible for crop losses not captured in this study, hence providing an aspect for future further research.

## Limitations and further research

The findings of this study would have been more generalizable if the number of farmers that constituted the sample size was more in the number. The complexities and systematic measurement errors in self-reported crop production data from smallholders in Africa identified by past studies^59–61^ are hereby acknowledged as limiting the findings of this study. The exclusion of temperature as a variable in this study is an obvious limitation, which would have provided improved outcome for drought assessment, especially with the use of SPEI, instead of SPI. Also, the estimation of a 6-month time-step for SPI (SPI-6) was omitted from the study, which could further strengthen findings. However, future studies can address these deficiencies, in addition to conducting a more detailed analysis of specific crops’ growth stages with climatic variables, and the impact of the use of spate irrigation systems, for more robust outcomes. Spate irrigation was not considered a priori as a factor that could affect the impacts of droughts in the area, hence further inquiry was not considered in this regard.

## Supplementary Information

Supplementary Information.

## Data Availability

The datasets used for this study are available and can be provided by the corresponding author on request.

## References

[1] Eze E, Girma A, Zenebe AA, Zenebe G (2020). Feasible crop insurance indexes for drought risk management in Northern Ethiopia. Int. J. Disaster Risk Reduct..

[2] Negash E, Gebresamuel G, Embaye T, Zenebe A (2019). The effect of climate and land-cover changes on runoff response in Guguf spate systems, northern Ethiopia. Irrig. Drain..

[3] Bayissa YA (2018). Developing a satellite-based combined drought indicator to monitor agricultural drought: A case study for Ethiopia. GIScience Remote Sens..

[4] Wilhite, D. A. Drought as a Natural Hazard: Concepts and Definitions. in *Drought: A Global Assessment* (ed. Wilhite, D. A.) 3–18 (Routledge, 2000). digitalcommons.unl.edu/droughtfacpub/69

[5] Viste E, Korecha D, Sorteberg A (2013). Recent drought and precipitation tendencies in Ethiopia. Theor. Appl. Climatol..

[6] Mera GA (2018). Drought and its impacts in Ethiopia. Weather Clim. Extrem..

[7] Qu C, Hao X, Qu JJ (2019). Monitoring extreme agricultural drought over the horn of Africa (HOA) using remote sensing measurements. Remote Sens..

[8] FDRE. *Multi-Sector Investment Plan for Climate Resilient Agriculture and Forest Development 2017–2030*. (2017).

[9] Gray C, Mueller V (2012). Drought and population mobility in rural Ethiopia. World Dev..

[10] Gebrehiwot T, Van Der Veen A, Maathuis B (2016). Governing agricultural drought: Monitoring using the vegetation condition index. Ethiop. J. Environ. Stud. Manag..

[11] Marteau R (2011). The onset of the rainy season and farmers’ sowing strategy for pearl millet cultivation in Southwest Niger. Agric. For. Meteorol..

[12] Mugalavai EM, Kipkorir EC, Raes D, Rao MS (2008). Analysis of rainfall onset, cessation and length of growing season for western Kenya. Agric. For. Meteorol..

[13] Nwite JN, Okolo CC (2016). Soil water relations of an Ultisol amended with agro-wastes and its effect on grain yield of maize (*Zea Mays* L.) in Abakaliki, Southeastern Nigeria. Eur. J. Sci. Res..

[14] Sivakumar, M. V. K. Agricultural Drought Indices. in *WMO/UNISDR Expert Group Meeting on Agricultural Drought Indices* (eds. Sivakumar, M. V. K., Motha, P. R., Wilhite, A. D. & Wood, A. D.) 1–197 (2011).

[15] Wubeneh NG, Sanders JH (2006). Farm-level adoption of sorghum technologies in Tigray, Ethiopia. Agric. Syst..

[16] Suryabhagavan KV (2017). GIS-based climate variability and drought characterization in Ethiopia over three decades. Weather Clim. Extrem..

[17] Gidey E, Dikinya O, Sebego R, Segosebe E, Zenebe A (2018). Analysis of the long-term agricultural drought onset, cessation, duration, frequency, severity and spatial extent using Vegetation Health Index (VHI) in Raya and its environs, Northern Ethiopia. Environ. Syst. Res..

[18] Warner, J. M. & Mann, M. L. Agricultural impacts of the 2015/2016 drought in Ethiopia using high-resolution data fusion methodologies. in Handbook of Climate Change Resilience (ed. Filho, W. L.) 1–26 (Springer International Publishing AG, 2018). 10.1007/978-3-319-71025-9_79-1.

[19] Funk, C. C. et al. A Quasi-Global Precipitation Time Series for Drought Monitoring. (2014).

[20] Shukla, S. et al. The Climate Hazards group InfraRed Precipitation with Stations (CHIRPS) dataset and its applications in drought risk management. Geophys. Res. Abstr. **19** (2017).

[21] Bayissa, Y. A. Developing an Impact-Based Combined Drought Index for Monitoring Crop Yield Anomalies in the Upper Blue Nile Basin , Ethiopia. (Delft University of Technology, 2018).

[22] Gebrechorkos SH, Hulsmann S, Bernhofer C (2018). Evaluation of multiple climate data sources for managing environmental resources in East Africa. Hydrol. Earth Syst. Sci..

[23] Dinku T, Funk C, Peterson P, Maidment R, Tadesse T (2018). Validation of the CHIRPS satellite rainfall estimates over eastern Africa. Q. J. R. Meteorol. Soc..

[24] Peng J (2020). A pan-African high-resolution drought index dataset. Earth Syst. Sci. Data.

[25] WMO, W. M. O. Standardized Precipitation Index User Guide. (2012).

[26] McKee, T. B., Doesken, N. J. & Kleist, J. The Relationship of drought frequency and duration to time scales. in Applied Climatology 6 (1993).

[27] WMO & GWP. Handbook of Drought Indicators and Indices. (2016).

[28] Yildirak K, Selcuk-Kestel AS (2015). Adjusting SPI for crop specific agricultural drought. Environ. Ecol. Stat..

[29] Wu D, Qu JJ, Hao X (2015). Agricultural drought monitoring using MODIS-based drought indices over the USA Corn Belt. Int. J. Remote Sens..

[30] Kogan FN (1995). Application of vegetation index and brightness temperature for drought detection. Adv. Sp. Res..

[31] Kidwell, K. B. Global Vegetation Index User’s Guide. (1990).

[32] Thenkabail, P. S., Amage, M. S. D. N. & Smakhtin, V. U. The Use of Remote Sensing Data for Drought Assessment and Monitoring in Southwest Asia. (2004).

[33] Kourouma JM (2021). Assessing the spatio-temporal variability of NDVI and VCI as indices of crops productivity in Ethiopia: A remote sensing approach. Geomatics. Nat. Hazards Risk.

[34] Jenkins DG, Quintana-Ascencio PF (2020). A solution to minimum sample size for regressions. PLoS ONE.

[35] Eze E, Girma A, Zenebe A, Kourouma JM, Zenebe G (2020). Exploring the possibilities of remote yield estimation using crop water requirements for area yield index insurance in a data-scarce dryland. J. Arid Environ..

[36] Elhag KM, Zhang W (2018). Monitoring and assessment of drought focused on its impact on sorghum yield over Sudan by using meteorological drought indices for the period 2001–2011. Remote Sens..

[37] Clarke, D. Assessing the impacts of drought on UK wheat production. **549**, (Cranfield University, 2017).

[38] Gunst, L. *et al.* Links between meteorological drought indices and yields (1979-2009) of the main European crops. *Wageningen University, Wageningen, The Netherlands* https://www.researchgate.net/publication/297758320_Links_between_meteorological_drought_indices_and_yields_1979-2009_of_the_main_European_crops (2015).

[39] Marshall, M. T., Funk, C. & Michaelsen, J. Agricultural drought monitoring in kenya using evapotranspiration derived from remote sensing and reanalysis data. in Remote Sensing of Drought: Innovative Monitoring Approaches (eds. Wardlow, B. D., Martha C. Anderson & Verdin, J. P.) 30 (CRC Press/Taylor & Francis, 2012).

[40] Shukla S, Mcnally A, Husak G, Funk C (2014). A seasonal agricultural drought forecast system for food-insecure regions of East Africa. Hydrol. Earth Syst. Sci..

[41] Cohen, J., Cohen, P., West, S. G. & Aiken, L. S. Applied Multiple Regression/Correlation Analysis for the Behavioral Sciences. (Lawrence Erlbaum Associates, 2003).

[42] Bachmair S, Tanguy M, Hannaford J, Stahl K (2018). How well do meteorological indicators represent agricultural and forest drought across Europe?. Environ. Res. Lett..

[43] Jones, J. R. et al. Establishing an index insurance triggers for crop loss in Northern Ghana. (2011).

[44] Zhu N, Xu J, Li W, Li K, Zhou C (2018). A comprehensive approach to assess the hydrological drought of Inland River Basin in Northwest China. Atmosphere (Basel).

[45] Blauhut V (2016). Estimating drought risk across Europe from reported drought impacts, drought indices, and vulnerability factors. Hydrol. Earth Syst. Sci..

[46] Yahaya M, Timothy DA (2015). Climate change and its effect on grain crops yields in the middle belt in Nigeria. Afr. J. Environ. Sci. Technol..

[47] WFP. Drought in Ethiopia: 10 Million People In Need. (2016).

[48] Haile GG (2019). Droughts in East Africa: Causes, impacts and resilience. Earth-Sci. Rev..

[49] FAO. Food security information for action: Practical guides. (2008).

[50] Peng, W. & Berry, E. M. The concept of food security. Encyclopedia of Food Security and Sustainability 1–7 (2019).

[51] Molla MA, Fitsume YD (2017). Seasonal rainfall and crop productivity in South West Ethiopia: Preliminary analysis for recent climate. Int. J. Water Resour. Environ. Eng..

[52] Legesse G, Suryabhagavan KV (2014). Remote sensing and GIS based agricultural drought assessment in East Shewa Zone, Ethiopia. Trop. Ecol..

[53] Legesse G (2010). Agricultural Drought Assessment using Remote Sensing and GIS Techniques.

[54] Elagib NA, Elhag MM (2011). Major climate indicators of ongoing drought in Sudan. J. Hydrol..

[55] Melese A, Suryabhagavan KV, Balakrishnan M (2018). Multimodel and vegetation indices for drought vulnerability assessment: A case study of afar region in Ethiopia. Remote Sens. Land..

[56] Li R, Tsunekawa A, Tsubo M (2014). Index-based assessment of Agricultural Drought in a Semi-arid Region of Inner Mongolia China. J. Arid Land.

[57] Madajewicz, M., Tsegay, A. H. & Lee, R. Managing risks in smallholder agriculture: The impacts of R4 on livelihoods in Tigray, Ethiopia from 2012 to 2016. (2017).

[58] Steenbergen FV, Mehari A, Alemehayu T, Almirew T, Geleta Y (2011). Status and potential of spate irrigation in Ethiopia. Water Resour. Manag..

[59] Abay KA, Abate GT, Barrett CB, Bernard T (2019). Correlated non-classical measurement errors, “second best” policy inference and the inverse size-productivity relationship in agriculture. J. Dev. Econ..

[60] Gourlay, S., Kilic, T. & Lobell, D. Could the debate be over? Errors in farmer-reported production and their implications for inverse scale–productivity relationship in Uganda. (2017). 10.1596/1813-9450-8192.

[61] Carletto C, Jolliffe D, Banerjee R (2015). From tragedy to renaissance: Improving agricultural data for better policies. J. Dev. Stud..

