# Supplementary material for: Predictors of drought-induced crop yield/losses in two agroecologies of southern Tigray, Northern Ethiopia
Source: Sci Rep. 2022 Apr 15;12:6284. doi: 10.1038/s41598-022-09862-x (PMC9012808; doi:10.1038/s41598-022-09862-x)
Supplement: Supplementary file 1 — Supplementary Information. [file 41598_2022_9862_MOESM1_ESM.docx]

**Appendix: Supplementary tables**

**Table S.01:** Regression coefficients predicting SCY, YLR and YpHa for 2015

| Location | Model | Results (Coefficients) | | Model | Results (Coefficients) | | Model | Results (Coefficients) | |
| --- | --- | --- | --- | --- | --- | --- | --- | --- | --- |
|  |  | Variable(s) | SCY (β) |  | Variable (s) | YLR (β) |  | Variable (s) | YpHa (β) |
| Entire area | 3 | (Constant)  KRFE2015  jun15_DNDVI  ND15 | -64.527  .157  -149.109  105.757 | 2 | (Constant)  KRFE2015  jun15_DNDVI | 295.572  -.897  468.785 | 1 | (Constant)  Annual_RFE | -113.684  .183 |
| Raya Azebo | 2 | (Constant)  Annual_RFE  July15_SPI3 | -133.234  .124  80.608 | 0 | ***No variables were entered into the model* |  | 2 | (Constant)  Annual_RFE  July15_SPI3 | -143.076  .134  87.755 |
| Endamekhoni | 1 | (Constant)  oct15_SPI12 | -1.088  -194.002 | 2 | (Constant)  sep15_SPI12  oct15_DNDVI | -233.671  325.562  -331.157 | 2 | (Constant)  oct15_SPI12  Aug15_DNDVI | 1.458  -240.513  44.231 |

**Table S.02:** Regression coefficients predicting SCY, YLR and YpHa for 2017

| Location | Model | Results (Coefficients) | | Model | Results (Coefficients) | | Model | Results (Coefficients) | |
| --- | --- | --- | --- | --- | --- | --- | --- | --- | --- |
|  |  | Variable (s) | SCY (β) |  | Variable (s) | YLR (β) |  | Variable (s) | YpHa (β) |
| Entire area | 2 | (Constant)  ND17  jul17_SPI12 | -58.444  156.706  40.771 | 2 | (Constant)  KRFE2017  sep17_SPI3 | 185.039  1.160  -1087.97 | 1 | (Constant)  jul17_SPI12 | -8.754  61.540 |
| Raya Azebo | 7 | (Constant)  ND17  jun17_DNDVI  aug17_DNDVI  jul17_DNDVI  oct17_DNDVI | -78.447  348.824  -314.265  -137.483  87.872  -51.776 | 1 | (Constant)  KRFE2017 | -735.855  1.375 | 7 | (Constant)  sep17_DNDVI  jun17_DNDVI  aug17_DNDVI  june17_SPI12  DNY2017 | -143.327  -52.981  -292.163  -85.806  -483.187  558.930 |
| Endamekhoni | 2 | (Constant)  sep17_DNDVI  KSDN17 | 11.250  167.665  29.275 | 1 | (Constant)  sep17_SPI3 | 737.378  -904.570 | 1 | (Constant)  sep17_DNDVI | 19.650  215.363 |
